# A comparison of the real-time controllability of pattern recognition to conventional myoelectric control for discrete and simultaneous movements

**DOI:** 10.1186/1743-0003-11-5

**Published:** 2014-01-10

**Authors:** Aaron J Young, Lauren H Smith, Elliott J Rouse, Levi J Hargrove

**Affiliations:** 1 Center for Bionic Medicine at the Rehabilitation Institute of Chicago, Chicago, IL, USA; 2 Department of Biomedical Engineering at Northwestern University, Evanston, IL, USA; 3 Feinberg School of Medicine at Northwestern University, Chicago, IL, USA; 4 Department of Physical Medicine and Rehabilitation at Northwestern University, Chicago, IL, USA

## Abstract

Myoelectric control has been used for decades to control powered upper limb prostheses. Conventional, amplitude-based control has been employed to control a single prosthesis degree of freedom (DOF) such as closing and opening of the hand. Within the last decade, new and advanced arm and hand prostheses have been constructed that are capable of actuating numerous DOFs. Pattern recognition control has been proposed to control a greater number of DOFs than conventional control, but has traditionally been limited to sequentially controlling DOFs one at a time. However, able-bodied individuals use multiple DOFs simultaneously, and it may be beneficial to provide amputees the ability to perform simultaneous movements. In this study, four amputees who had undergone targeted motor reinnervation (TMR) surgery with previous training using myoelectric prostheses were configured to use three control strategies: 1) conventional amplitude-based myoelectric control, 2) sequential (one-DOF) pattern recognition control, 3) simultaneous pattern recognition control. Simultaneous pattern recognition was enabled by having amputees train each simultaneous movement as a separate motion class. For tasks that required control over just one DOF, sequential pattern recognition based control performed the best with the lowest average completion times, completion rates and length error. For tasks that required control over 2 DOFs, the simultaneous pattern recognition controller performed the best with the lowest average completion times, completion rates and length error compared to the other control strategies. In the two strategies in which users could employ simultaneous movements (conventional and simultaneous pattern recognition), amputees chose to use simultaneous movements 78% of the time with simultaneous pattern recognition and 64% of the time with conventional control for tasks that required two DOF motions to reach the target. These results suggest that when amputees are given the ability to control multiple DOFs simultaneously, they choose to perform tasks that utilize multiple DOFs with simultaneous movements. Additionally, they were able to perform these tasks with higher performance (faster speed, lower length error and higher completion rates) without losing substantial performance in 1 DOF tasks.

## Background

Myoelectric prostheses use surface electromyography (EMG) signals to direct the movement of prosthesis degrees of freedom (DOFs) (e.g. elbow flexion, wrist rotation, etc.), and are a common method for treating upper-extremity amputation. Ideally, the control of such devices allows for an intuitive replication of the lost limb’s function. One component of natural limb control that is not adequately replaced by current myoelectric technology is the ability to control multiple DOFs simultaneously. Simultaneous control of multiple joints is frequently used in the able-bodied control of the upper extremity during activities of daily living, such as wrist rotation and grasp to turn a door knob. The mechanical means for supplying such functionality to a prosthesis have been addressed by the recent development of multifunction hands [1,2] and advanced arm prototypes [3,4]. However, there are currently no myoelectric-control algorithms that have demonstrated successful simultaneous control in amputee populations during real-time, functional evaluations.

Conventional amplitude-based control (referred to in this paper as conventional control) of a myoelectric prosthesis refers to comparing an amplitude estimate of the EMG signal to a set of predetermined thresholds. The most intuitive form of conventional control uses EMG amplitude estimates recorded by electrodes at two ‘control sites’, which are ideally placed over a physiologically appropriate (when possible) antagonistic muscle pair in the residual limb to control a single DOF [5]. It is important that muscle pair be independently controlled and free of EMG crosstalk. In most upper extremity amputees, there are not enough independent control sites for simultaneous control – due to either a lack of independently controlled muscles in the residual limb (transhumeral amputees) or significant EMG crosstalk between electrode sites on the forearm (transradial amputee) [6]. Mode switches such as force sensing resistors (FSRs) or muscle co-contraction are used to sequentially control multiple DOFs [5].

Targeted muscle reinnervation (TMR) surgery is a recently developed surgical technique that restores physiologically appropriate, independent control sites to high-level amputees [7]. The surgical technique redirects residual nerves to muscles that are redundant or have no biomechanical function after amputation. After typical surgeries for transhumeral [8] or shoulder disarticulation [9] amputees, at least four independent myoelectric control sites are available and have been used to provide simultaneous control of 2 DOFs using conventional control methods [9,10]. This surgical technique was first performed as a research procedure on a patient in 2002 but has now performed as a clinical procedure. We and our collaborators are aware of this procedure being performed in over 60 high-level upper limb amputee patients.

Pattern recognition approaches currently in development [11-13] address many limitations of the conventional control approach. Pattern recognition uses classifiers trained on labelled EMG signal exemplars to discriminate between multiple classes of intended motions [11]. Pattern recognition methods do not require independently, crosstalk free control sites, nor do they require mode-switching. EMG signals are typically recorded from physiologically appropriate muscles given the set of motion classes to control. A wide variety of feature set and classifier combinations have been shown to provide excellent classification performance, but nearly all implementations only provide seamless sequential control of multiple DOFs [14]. Linear discriminant analysis (LDA) [15] using time domain [11] and autoregressive [16] features of the EMG signal are frequently employed because they the system is easy to train and computationally efficient. The performance of such algorithms have been compared to conventional control in healthy control subjects [17] and amputees [18], where pattern recognition outperforms conventional control in experienced users.

Pattern recognition-based algorithms also have the potential to improve simultaneous control, as it is not limited to TMR populations. Pattern recognition algorithms using LDA classifiers have been successfully extended to provide simultaneous multi-DOF control in healthy control subjects [19] and non-human primates [20]. Both the standard approach using a single LDA classifier trained to recognize simultaneous movements [21] and more complex organizations of multiple LDA classifiers [20,22] have been evaluated. However, to date there have been no online functional tests of pattern recognition-based simultaneous control in either healthy control subjects or amputees.

The main objective of this work was to determine if using a control system that enabled simultaneous control would maintain performance on tasks that required control over only a single DOF and improve performance on tasks that required simultaneous control over 2 DOFs. Secondary objectives were 1) to compare sequential pattern recognition control to conventional amplitude control and 2) to compare simultaneous pattern recognition control to conventional amplitude control. Subjects were amputees who had undergone TMR surgery, which are the only amputee population where simultaneous conventional control may be employed. We report on the performance of TMR subjects in controlling a virtual reality prosthesis during a real-time interactive tasks.

## Methods

### Experimental protocol

Four subjects who had undergone TMR [7] completed the following experiment that had been approved by the Northwestern University Institutional Review Board after giving their written informed consent. Two subjects were shoulder disarticulation amputees and two subjects were transhumeral amputees (see Table 1). All four subjects had reinnervated muscle sites for controlling hand open and hand close movements, and the shoulder disarticulation subjects also had reinnervated sites for controlling elbow flexion and elbow extension movements. For transhumeral subjects, the elbow flexion and extension sites were controlled by the natively innervated heads of the biceps and triceps brachii. Each of the patients had experience using conventional control of these four sites with a myoelectric prosthesis used at home. Self-adhesive silver/silver chloride bipolar surface electrodes were used to record muscular activity (Noraxon Dual electrodes) with a 1 cm diameter conductive area and a 2 cm interelectrode distance. Four pairs of electrodes were placed directly over the subjects’ control sites that they used to control their conventional myoelectric prostheses. Additional pairs of electrodes were placed adjacent to the primary sites, located as areas with palpable muscle activity. Electrodes were placed to avoid the nipple, locations over excess subcutaneous adipose/breast tissue [23], and locations susceptible to sweat (e.g. near the axilla). Shoulder disarticulation subjects had six to eight additional electrodes, while transhumeral subjects (which had decreased surface area for electrode placement), had four additional electrodes. Therefore, a total of eight to twelve EMG channels were used. A ground electrode was placed on the bony prominence of the shoulder.

**Table 1:** Subject specific details

**Subject**	**Amputation type**	**Time since amputation (years)**	**Time since TMR surgery (years)**
1	Transhumeral	6	6
2	Transhumeral	3	3
3	Shoulder disarticulation	11	10
4	Shoulder disarticulation	7	7

Signals were amplified by a factor of 800× using research electrodes from Liberating Technologies, Inc. (Boston, MA) and a second gain stage multiplied the signal amplitude by an additional 6×. Signals were digitally sampled at 1000 Hz and high pass filtered at 20 Hz using a 3rd order Butterworth filter to reduce motion artifact and acquired using a custom 16-bit data acquisition system.

Amputees were prompted to perform eight different motions using the screen training method described previously [24]. These motions were: elbow flexion (EF), elbow extension (EE), hand close (HC), hand open (HO), elbow flexion in combination with hand open (EF/HO), elbow flexion with hand close (EF/HC), elbow extension with hand open (EE/HO), and elbow extension with hand close (EE/HC), where the first four are referred to as discrete movements and the second four as combined movements. Internally developed software – Control Algorithms for Prosthetics (CAPS) [7] – was used to prompt each motion a total of four times for 3 seconds with a 3 second rest between each motion. The subjects were instructed to make medium, constant force contractions to the best of their ability and no feedback was provided to assist subjects during the data collection procedure.

EMG data were divided into 250 ms windows with a 50 ms frame increment [13,25]. These windowing parameters have been shown to provide a good tradeoff between controller delay and classification error during real-time control [25]. For pattern recognition-based techniques, four time-domain features (mean absolute value, number of zero crossing, number of slope sign changes, and waveform length) [11] and six autogressive features (the six coefficients of a sixth order autoregressive model) [16] were extracted from the EMG windows and a linear discriminant analysis was used for classification.

**Figure 1:**
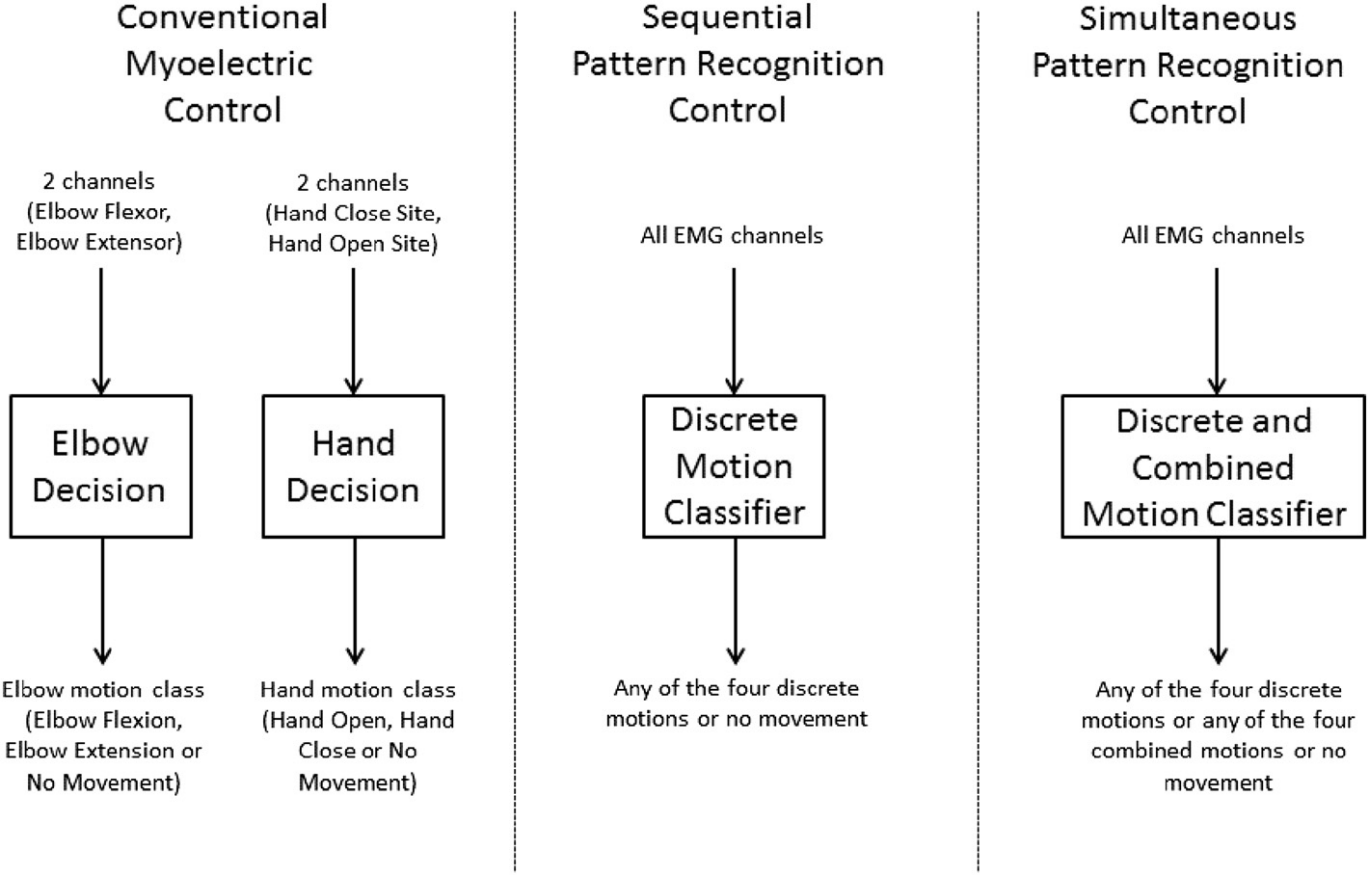
**Block diagram of the three control strategies.** Conventional myoelectric control (left) uses two separate decision nodes, each receiving two channels to determine a motion output based on EMG amplitude. Sequential pattern recognition control receives all EMG channels as input and can output any of the four discrete motions (Elbow Flexion/Extension or Hand Open/Close) or no movement. Simultaneous pattern recognition control also receives all EMG channels as input, outputs any of the four discrete motions, no movement, and additionally, any combination of elbow and hand movements.

Functional tests were performed on three control strategies in this study (see Figure 1). Ample rest breaks were taken during and between each condition to help prevent fatigue; previous studies have also shown that the effects of muscle fatigue on classification accuracy for surface EMG are minimal [26]. The first strategy, conventional amplitude based control (or ‘conventional control’), was configured by a certified prosthetist to be the same control system as the subject’s myoelectric prosthesis used at home. This condition served as the control for this study. Amputees controlled EF, EE, HC, and HO motions using their four independent muscle sites. Since these sites are independent in patients with TMR, the subjects were capable of performing simultaneous control of elbow and hand movements using this control configuration [9].

The second control strategy, referred to as ‘sequential’ pattern recognition based control (or ‘sequential control’), is the pattern recognition method previously reported in the literature [13] where DOFs are activated one at a time. While simultaneous control is not possible with previously implemented pattern recognition techniques, seamless transitions are possible between DOFs potentially allowing for quick movements through multiple DOFs to reach a target [17]. For the sequential control condition, the discrete movements (EF, EE, HC, and HO) were trained and controlled in real-time testing trials.

The third control strategy was simultaneous pattern recognition based control (or ‘Simultaneous control’) that has been tested offline before in able-bodied subjects [22]. This previous study, which compared multiple simultaneous classification methods, showed that a single LDA classifier performed well for two DOF control; and thus was selected for the present study for its simplicity and efficiency. In this condition, simultaneous movements of the elbow DOF with the hand DOF are made possible by training the pattern recognition algorithm with signals generated from amputees attempting to perform simultaneous movements. Each combined and discrete motion was trained as a separate class in this condition for a total of eight classes or movement types (four combined and four discrete).

**Figure 2:**
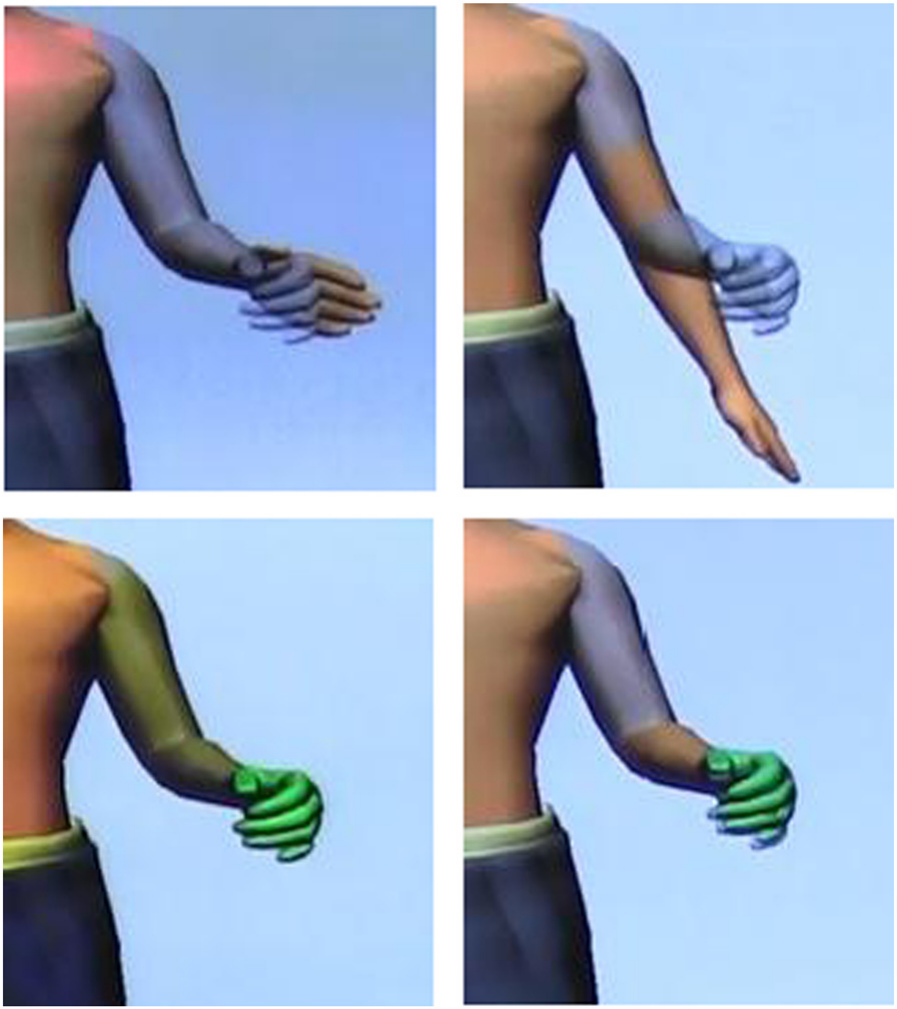
**Target Achievement Control (TAC) test.** Start (top) and end (bottom) of a discrete movement of closing the hand (left) and combined movement of elbow flexion and hand close (right).

Functional testing of the three conditions was performed in a virtual environment using the Target Achievement Control (TAC) Test [27]. For the TAC test, the virtual prosthesis started in a non-neutral position and were instructedto move the virtual prosthesis into the target posture was located in the middle of the virtual workspace (see Figure 2). Visual feedback was provided such that the virtual arm turned green when it was within an acceptable tolerance of the target (+/- 15° for each DOF), and the test was completed when the subject held the virtual arm within the target for a duration of 1 s. If the subject overshot the target or produced undesired movements, necessary corrections had to be made to reach the target. A total time of 20 s was given per trial to complete the task. The speed was proportionally controlled based on the mean absolute value of EMG signals across all the channels, normalized for each subject for a specific motion class. Combined motion classes speed was calculated in the same way, and the calculated speed was used for both DOFs. Thus, both DOFs in a combined movement had the same speed relative to each other. A velocity dependent ramp of 500 ms [28] was used to reduce the effects of spurious misclassifications on performance.

Subjects completed the same real-time testing protocol for all three conditions – conventional control, sequential control, and simultaneous control. TAC tests that required movement through just 1 DOF were completed first, thus only discrete movements were needed to complete these tests, but for conventional and simultaneous controllers, combined movements could be accidently performed and cause errors. Each of the four discrete movements were prompted five times for a total of 20 discrete movement trials for each control strategy. Next, TAC tests that required movement in two DOFs were conducted and similarly each of the four combined movements were prompted five times for a total of 20 combined movement trials for each control strategy. In these trials, subjects could complete the task more efficiently if they performed a correct simultaneous movement directly toward the target, but could still complete the trial using purely sequential movements.

With two subjects, the simultaneous pattern recognition control strategy was tested qualitatively using a physical prosthesis. No performance metrics were acquired, but functional tasks using simultaneous movements were conducted to demonstrate the ability of the controller to perform simultaneous movements in activities of daily living. An accompanying videos (Additional files 1 and 2) of the amputees using the simultaneous pattern recognition control strategy is included.

### Data analysis

The data for each trial of the TAC test were segmented to begin from the time when movement was initiated until the time the subject entered the target (including tolerances) and remained there until the completion of the trial. From these segmented data, each of the metrics were determined, and used to quantify subject performance.

#### Completion time and completion rate

Completion rate and completion time quantify the performance of subjects ability to complete the TAC test trials in a timely manner and are the primary metrics for analyzing the functional performance of a subject during real-time tests [27]. The completion time was defined as the time from movement initiation to the completion of the trial. The completion rate is a function of time was defined as the percentage of total trials completed before a given amount of time.

#### Length error

The ability of the subject to efficiently control the simulated DOFs during the TAC test as quantified by the length error percentage. This metric is defined as the length the subject traversed beyond the total required distance, as a percentage of the total required distance. The total required distance was defined as the straight-line distance between the starting configuration and the target configuration, not including any distance traveled within the target itself. Thus, a length error of zero percent indicates a perfect trial and an error of 100% indicates the subject’s path length was twice the total required distance.

#### Active DOFs

The ‘Active DOFs’ metric quantified how the subject utilized the available DOFs to complete each trial. The metric is defined by the percentage of time that zero, one or two DOFs were moving during each trial. For example, the sequential control scheme prevents the movement of more than a single DOF, therefore this controller is constrained to show movement in only zero and one DOFs.

#### Statistical analysis

Statistical analysis was performed for each performance metric (classification error, completion time, completion rate at 5 seconds and length error). For each metric, an ANOVA was performed with the performance metric as the response variable, subject as a random factor, number of DOFs in the task (1 or 2), control strategy, and the interaction between number of DOFS in the task and control strategy as fixed factors. For each ANOVA, each significant factor was reported at the α = 0.05 level.

## Results

### Offline classification results

Overall offline classification errors (percent of overall decisions that were misclassified as the wrong motion class) for individual subjects for the pattern recognition control strategies are shown in Tables 2 and 3. In the ANOVA, classification error was significant (p < 0.05) between groups (sequential, simultaneous for discrete motions and simultaneous with combined motions), subject was also significant (p < 0.05). Average classification error for sequential control was 11.1% (+/- 5.8 SEM) and error for simultaneous pattern recognition control was 23.1% (+/- 10.3 SEM) and 33.19% (+/- 11.3 SEM) for discrete and combined movement classification, respectively.

**Table 2:** Individual performance results (1 DOF complexity)

**Condition**	**Subject**	**Completion time (s)**	**Completion rate (%)**	**Length error (%)**	**Classification error (%)**
Conventional	1	3.6 (1.73)	75	190.1 (99.27)	-
	2	2.3 (1.65)	90	20.7 (17.90)	-
	3	1.4 (0.49)	100	16.3 (8.51)	-
	4	3.3 (2.76)	80	162.5 (198.32)	-
Sequential	1	1.1 (0.16)	100	12.1 (0.86)	2.5
	2	1.7 (0.38)	100	11.7 (0.26)	1.3
	3	1.3 (0.30)	100	17.0 (10.80)	26.1
	4	1.5 (0.36)	100	14.9 (6.66)	14.3
Simultaneous	1	2.4 (1.29)	85	46.3 (50.29)	8.6
	2	1.3 (0.21)	100	11.8 (0.24)	6.5
	3	1.9 (0.53)	95	32.6 (21.96)	51.1
	4	2.5 (1.27)	95	37.3 (45.57)	26.3

**Table 3:** Individual performance results (2 DOF complexity)

**Condition**	**Subject**	**Completion time (s)**	**Completion rate (%)**	**Length error (%)**	**Classification error (%)**
Conventional	1	4.4 (2.54)	65	124.2 (97.27)	-
	2	3.5 (1.39)	90	94.0 (55.58)	-
	3	2.2 (0.72)	100	44.9 (16.27)	-
	4	4.1 (1.74)	70	83.5 (41.94)	-
Sequential	1	2.9 (0.29)	95	67.6 (16.76)	-
	2	4.1 (0.68)	90	63.7 (10.02)	-
	3	3.5 (0.37)	90	67.4 (6.40)	-
	4	3.9 (0.34)	95	72.8 (14.02)	-
Simultaneous	1	1.4 (0.04)	100	7.9 (7.68)	17.1
	2	1.9 (0.97)	100	25.6 (28.02)	12.3
	3	2.4 (1.30)	95	27.0 (16.93)	60.1
	4	2.0 (0.97)	100	27.0 (47.67)	43.3

### Statistical results for real-time performance measures

The control strategy – conventional, sequential or simultaneous – was a significant factor (p < 0.05) in the ANOVA results for completion time, completion rate (at 5 s), and length error. Additionally, the number of DOFs in the task was significant (p < 0.05) for completion time and the interaction between number of DOFs in the task and control strategy was significant (p < 0.05) for completion time. Interpretations of these results and comparisons between each of the control strategies are made below.

### Conventional vs. Sequential control

**Figure 3:**
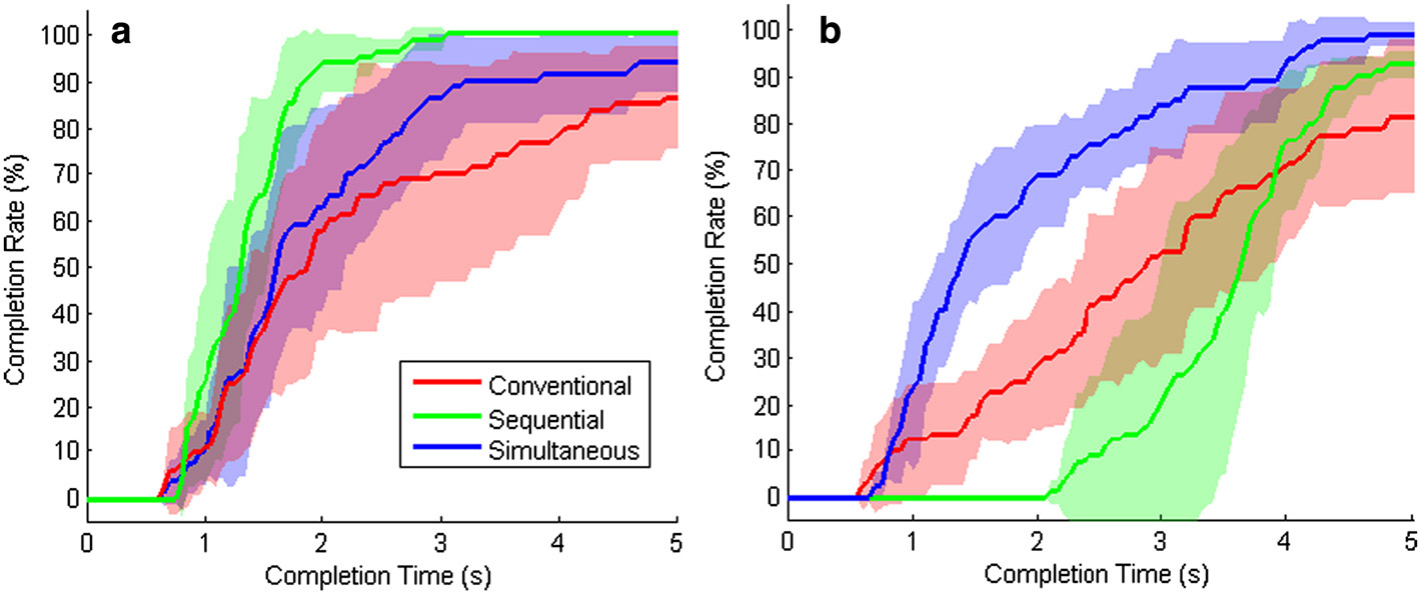
**TAC test completion rate verse completion time for the three control strategies.** These figures show the completion rate as a function of completion time across all four subjects for each of the three control strategies. The shaded regions indicate +/- 1 SEM for each of the lines. Figure 3**a** is for tasks with 1 DOF complexity, while Figure 3**b** is for tasks with 2 DOF complexity.

**Figure 4:**
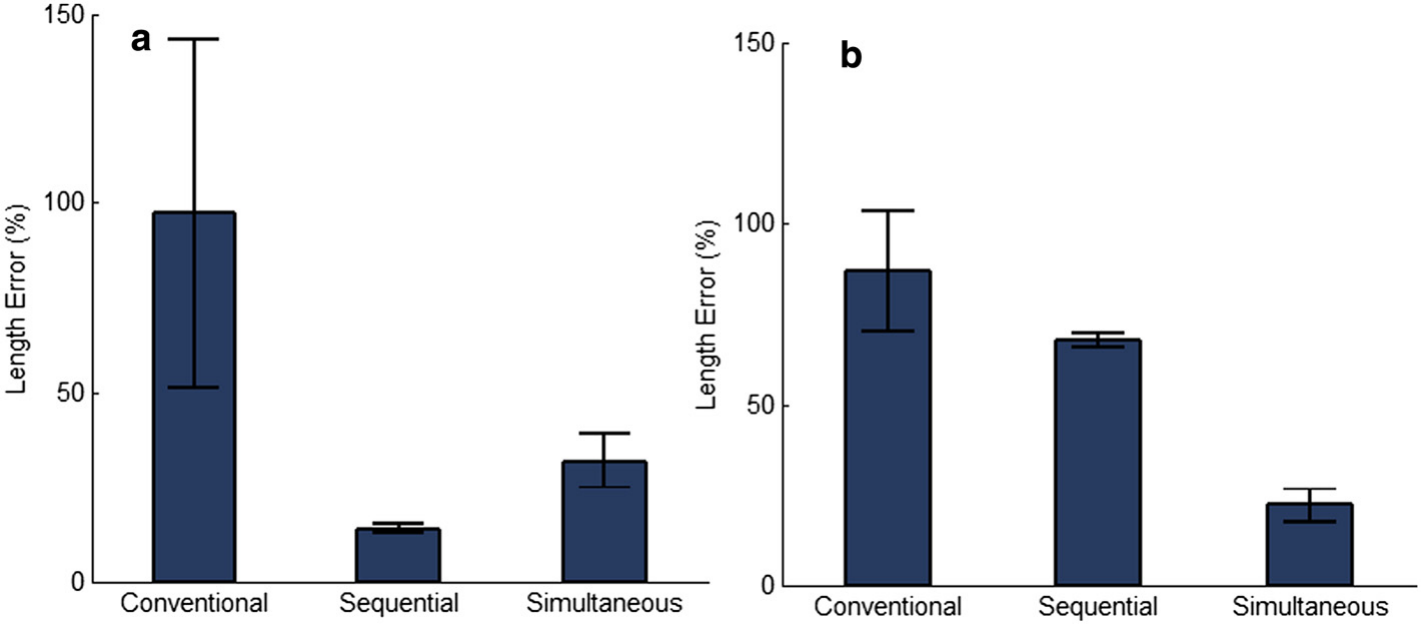
**TAC test length error for each of the three control strategies.** These figures show the % deviation from the perfect path averaged across all four subjects for each of the three control strategies. Figure 4**a** is for tasks with 1 DOF complexity, while Figure 4**b** is for tasks with 2 DOF complexity.

In 1 DOF tasks, subjects had higher performance using sequential control compared to conventional control (see Table 2). The curve in Figure 3a shows subjects completed tasks faster with a higher completion rate at 5 seconds using sequential control compared to conventional control. Additionally, the length error (Figure 4a) was less for sequential control compared to conventional control for 1 DOF tasks. Lastly, subjects using conventional control completed the tasks by moving zero, one and two DOFs while subjects using sequential control moved only one DOF.

**Figure 5:**
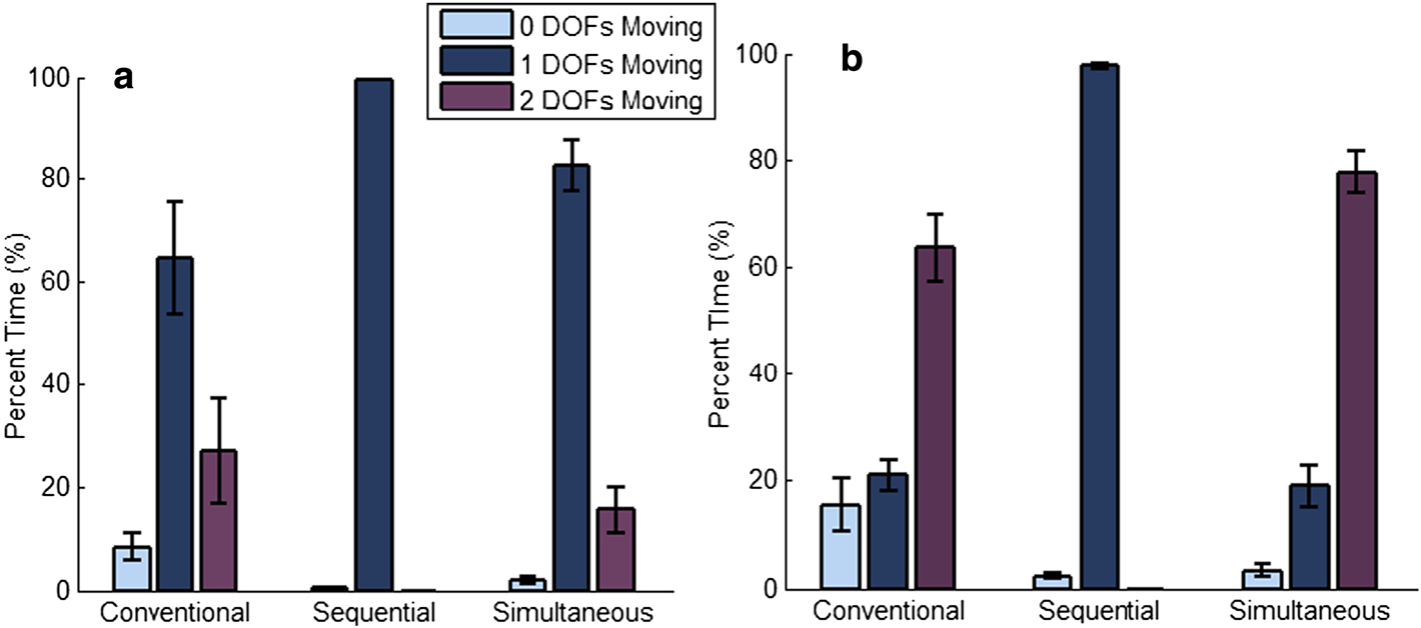
**Percent of time in which zero, one, or two DOFs were moving for each control strategy.** Figure 5**a** shows the average percent of time across the four subjects in which zero, one or two DOFs were moving for tasks of 1 DOF complexity for each of the three control strategies, and Figure 5**b** is similar except for 2 DOF complexity.

Alternatively, for 2 DOF tasks, subjects using conventional control completed more trials before the 4 s time point compared to sequential control (Figure 3b), but more trials were completed by sequential control after the 4 s time point. Furthermore, sequential control had a lower length error on 2 DOF tasks (Figure 4b), similar average completion times, and higher average completion rates (Tables 2 and 3) compared to convention control. For 2 DOF tasks using conventional control, subjects chose to use two DOF movements 64% of the time (Figure 5b), which was not possible with sequential control and likely accounted for the increased completion times before the 4 s time point compared to sequential control.

### Conventional control vs. Simultaneous pattern recognition control

For 1 DOF tasks, simultaneous pattern recognition control demonstrated superior performance compared to conventional control based on metrics in Table 2. In Figures 3a and 4a, subjects completed trials faster with lower length errors using simultaneous control compared to conventional control. Additionally, based on Figure 5a, subjects simultaneously controlled two DOFs slightly more frequently using conventional control.

For 2 DOF tasks, the simultaneous pattern recognition control demonstrated superior performance (Tables 2 and 3). Figures 3b and 4b show that subjects completed trials faster with lower length errors with simultaneous control when compared to conventional control. Based on Figure 5b, in both conditions, subjects chose to use simultaneous movements for the majority of the time: 78% for simultaneous and 64% for conventional control.

### Sequential vs. Simultaneous pattern recognition control

The sequential pattern recognition controller performed better on 1 DOF tasks than the simultaneous pattern recognition controller (Tables 2 and 3), as sequential controller had lower completion time (Figure 3a) and length error (Figure 4a) compared to simultaneous control for 1 DOF tasks. However, for 2 DOF tasks, the simultaneous pattern recognition controller had better performance across all metrics compared to the sequential controller (Tables 2 and 3). This is demonstrated in both Figures 3b and 4b with faster completion times and lower length error for 2 DOF tasks.

## Discussion

The joint angles resulting from a single shoulder disarticulated TMR subject attempting to simultaneously control a prosthetic elbow and hand have been presented previously [10]. That data was measured when the subject was required to move both DOFs freedom through their range of motions. During this experiment, the patients were required to complete performance tests that required movements of 1 or 2 DOFs while instructed to complete the task as quickly as possible (though not explicitly instructed to use simultaneous control). The main objective of this paper was to determine if using a control system that allowed simultaneous control would maintain performance on tasks that required control over only a single DOF and improve performance on tasks that required control of 2 DOFs. Our results suggest that a properly configured control system do allow these goals to be achieved.

Pattern recognition has been shown to have performance benefits over conventional control in able bodied subjects activating their wrist flexors/wrist extensors with mode switching for the conventional scheme [17]. Few direct comparisons between the real-time performance of conventional control and pattern recognition have been reported in amputees. A case study of a transradial amputee [29] showed that multifunctional hand control with sequential movements was slower and tended to perform worse on outcome metrics than mode switching using a conventional control strategy. However, the subject’s lack of familiarity with pattern recognition systems was cited as contributing significantly to these results. A direct comparison between simultaneous multifunctional pattern recognition control and conventional control in amputees has not been previously reported. Specifically, in this study, subjects that underwent TMR surgery were selected as this allows the conventional strategy to control 2 DOFs independently (no mode switches are necessary). This is one of the few clinically available uses of simultaneous control with myoelectric signals controlling all DOFs, and thus was an appropriate control to compare proposed simultaneous pattern recognition control strategies.

Traditional pattern recognition based control has been criticized for its inability to perform simultaneous movements, and while this study demonstrated this criticism (Figure 3b), sequential pattern recognition control still had performance benefits in 2 DOF tasks compared to conventional control (more than 10% higher completion rate at 5 s and lower length error). Additionally, sequential pattern recognition had higher performance in terms of all metrics tested compared to conventional control on 1 DOF tasks. Overall, the sequential pattern recognition control seems to be a functionally superior system for amputees compared to conventional control, even though it does not use simultaneous control.

In this study, we have extended pattern recognition based techniques to recognize simultaneous movements and have demonstrated functional improvements compared to conventional control for both single movement (1 DOF) tasks and combined movement (2 DOF) tasks. Amputees performed better with simultaneous pattern recognition control on 2 DOF tasks compared to conventional or sequential control, demonstrating the potential of such a system for clinical use. Additionally, the simultaneous pattern recognition controller only slightly underperformed the sequential controller on 1 DOF tasks and performed better than conventional control on 1 DOF tasks. These results suggest that simultaneous control is beneficial with pattern recognition without substantially sacrificing the advantages of sequential pattern recognition control. This is important as many real-world tasks involve the use of multiple DOFs at the same time across the elbow, wrist, and/or finger joints. With new advancements in upper limb prostheses, amputees can substantially benefit from controllers capable of providing simultaneous movements for everyday tasks; however, this ability is lost with sequential pattern recognition based control, and even the seamless transitions between DOFs possible with sequential control does not compensate for the lack of simultaneous control (Figure 3b).

An interesting result was the relatively high classification errors for the pattern recognition systems in some users (Tables 2 and 3) – e.g. Subject 3. While users with less classification error tended to have higher performance metrics, all subjects had high completion rates at the 5 s mark using pattern recognition strategies. Thus, even systems with high offline error were able to perform well functionally. We believe that the users were able to adapt contraction patterns when provided with real-time feedback to achieve the specified postures within the TAC test.

One important limitation of this study was that functional testing only occurred in a virtual environment. Matching target postures was able to show important differences between simultaneous myoelectric controllers, but tests on physical prostheses would likely elucidate advantages and disadvantages of simultaneous control in greater detail than virtual posture matching. The qualitative evaluation of the simultaneous pattern recognition control demonstrated in the accompanying video (Additional files 1 and 2) shows promise and the simultaneous pattern recognition appears smoother and more fluid. However, important future work with physical prosthesis comparing the differences between pattern recognition and conventional myoelectric control strategies will be a future focus.

Another issue with the simultaneous pattern recognition controller was the velocity control strategy used. While the subjects were capable of modulating the overall speed of a simultaneous movement, both DOFs travelled at the same speed during simultaneous movements. This may have provided an advantage for the simultaneous pattern recognition controller as targets could be reached by moving the virtual prosthesis at the same speed in both DOFs. However, this velocity control strategy had a significant disadvantage in that the speeds of DOFs could not be adjusted relative to each other to correct for mistakes. Future research should investigate strategies to proportionally control multiple DOFs independently from each other during which will likely provide amputees with an enhanced ability to perform simultaneous movements and further increase functional performance using simultaneous control strategies.

A final limitation was the low subject number (N = 4) recruited for this experiment. TMR is a surgical technique that was proposed in the previous decade, and the number of TMR recipients remains quite small although it continues to grow. This limited the number of subjects potentially available for this experiment. Due to the low subject number, the statistical analyses performed were limited and only very large differences reached significance. Some of the trends that were shown may have been significant (or not) with more subjects. The authors thought that this was a useful population of amputees to compare conventional control to pattern recognition techniques because this is the only group of upper limb amputees that can control multiple DOFs independently using conventional control. The results found in this experiment should be applicable to transradial amputees as similar sequential and simultaneous pattern recognition control strategies may be used with the exception that only single DOF (or multiple with mode switches) conventional control strategies may be used.

## Conclusions

The main objective of this study was to determine if using a control system that enabled simultaneous control would improve performance on tasks that required control over 2 DOF and maintain performance over tasks that required only a single DOF. In this study, we showed that on four amputees with TMR, this objective was met using a simultaneous pattern recognition controller – performance was only slightly less compared to sequential control on single DOF tasks and improved on 2 DOF tasks. Additionally, we demonstrated that amputees using sequential pattern recognition performed better on single DOF tasks than convention control, and similar or better on 2 DOF tasks. Thus the findings of this study indicate that pattern recognition based control is preferable for single movement tasks and should be extended to incorporate simultaneous movements for control over tasks involving more than a single movement.

## Competing interests

The authors declare that they have no competing interests.

## Authors’ contributions

AY helped in conceiving the study concept and design, acquiring the data, analyzing and interpreting the data, and drafting the manuscript. LS helped in conceiving the study concept and design, acquiring the data, drafting the manuscript, and critically revising for important intellectual content. ER helped in analyzing and interpreting the data and drafting the manuscript. LH helped in conceiving the study concept and design, drafting the manuscript, critically revising for important intellectual content, obtaining funding, and supervising the study. All authors read and approved the final manuscript.

## Supplementary Material

Additional file 1 **Simultaneous pattern recognition motions.** The subject controls all combinations of simultaneous elbow and hand motions (e.g. elbow up and hand closed simultaneously). This video demonstrates all possible simultaneous movements used in this study. Click here for file

Additional file 2 **Simultaneous pattern recognition control demonstration.** This video demonstrates a subject using simultaneous pattern recognition control during functional movements. The subject throws a tennis ball, catches a bag being thrown in the air, and picks up a rolling tennis ball. Note the fluidity of the subject’s motion. He coordinates opening/closing his hand, flexing his elbow, and swinging his shoulder. To contrast, the last video segment is the same subject picking up a rolling tennis ball using sequential pattern recognition control. The subject must use hand closed and then elbow up, leading to less fluid control. Click here for file
